# Potential Role of Vitamin B6 as an Antioxidant via Pyridoxal-5′-Phosphate–Dependent Metabolic Pathways and Subsequent Activation of Nrf2 Signaling

**DOI:** 10.3390/nu18101499

**Published:** 2026-05-08

**Authors:** Norihisa Kato, Yongshou Yang, Abdelkrim Khedara, Thanutchaporn Kumrungsee

**Affiliations:** 1 Graduate School of Integrated Sciences for Life, Hiroshima University, Higashi-Hiroshima 739-8528, Japan; kumrung@hiroshima-u.ac.jp; 2 School of Life Sciences, Anhui University, Hefei 230601, China; 21193@ahu.edu.cn; 3 Laboratory of Molecular and Cellular Biology, Faculty of Natural and Life Sciences, Constantine 1 Frères Mentouri University, Constantine 25017, Algeria; khedara.abdelkarim@umc.edu.dz

**Keywords:** vitamin B6, pyridoxal 5′-phosphate, antioxidant, Nrf2, carnosine, taurine, hydrogen sulfide, kynurenine pathway, neurotransmitter, glycogen phosphorylase

## Abstract

Accumulating evidence suggests that vitamin B6 (B6) deficiency among older adults is associated with sarcopenia, frailty, heart disease, and brain diseases. Oxidative stress and inflammation play key roles in cardiac and skeletal muscle and neuronal pathology. However, the detailed roles of B6 supplementation in oxidative stress and inflammation are not fully understood. Recent studies have shown that supplemental B6 upregulated the nuclear factor erythroid 2-like 2 (Nrf2) signaling pathway with the coordinated activation of antioxidant responses. Accumulating evidence suggests the potential of targeted Nrf2 signaling regulation in the treatment of aging-related musculoskeletal, heart, and brain diseases. Notably, dietary supplementation of B6 elevates the levels of several antioxidant metabolites, such as carnosine, anserine, taurine, hydrogen sulfide (H_2_S), 5-methyltetrahydrofolate, kynurenic acid, 3-hydroxyanthranilic acid, and γ-aminobutyric acid (GABA) via the upregulation of pyridoxal 5′-phosphate (PLP)-dependent metabolic pathways, thereby linking to Nrf2 signaling activation. Furthermore, supplemental B6 stimulates glycogen breakdown through the PLP enzyme, glycogen phosphorylase, which in turn enhances the pentose phosphate pathway, thereby increasing nicotinamide adenine dinucleotide phosphate (NADPH) availability to regenerate glutathione (GSH). In this perspective article, we propose the potential role of B6 as an antioxidant mediated by the PLP-dependent multi-metabolic productions of antioxidant metabolites.

## 1. Introduction

Vitamin B6 (B6) is a water-soluble vitamin found in various animal and plant foods. It is also available in fortified cereals and supplements. B6 has six isoforms, known as B6 vitamers: pyridoxine (PN), pyridoxal (PL), pyridoxamine (PM), and their phosphorylated forms. Among these, pyridoxal 5′-phosphate (PLP) is the most active and acts as a coenzyme in over 150 reactions. These reactions include amino acid synthesis, transformation, and degradation of amino acids; one-carbon unit provision; transsulfuration; synthesis of tetrapyrrolic compounds, polyamines, and hydrogen sulfide (H_2_S) synthesis; neurotransmitter biosynthesis and degradation; and glycogen breakdown [1]. Additionally, B6 itself has radical scavenging activity [1].

Although severe B6 deficiency is a rare condition, marginal B6 deficiency is common globally [2]. Generally, subclinical deficiency of B6 can precipitate biochemical changes that become more obvious over time as the deficiency progresses. Increasing evidence shows that B6 might exert protective effects against chronic diseases, such as cardiovascular diseases and cancers, by mitigating oxidative stress, inflammation, inflammasomes, and DNA instability [1,3]. Over recent decades, the global population has been aging rapidly. Research on maintaining physical function and metabolic health revealed that sarcopenia, a condition characterized by aging-related loss of muscle mass and strength, has become a critical problem in aging societies. Nutritional intervention therapy, which includes adequate intake of proteins, antioxidative and anti-inflammatory nutrients such as omega-3 fatty acids, and vitamin D, is the most prevalent protective approach for sarcopenia [4].

Recently, our study has highlighted that B6 deficiency is associated with the risks of sarcopenia and mortality in older adults [5]. Furthermore, accumulating evidence indicates that B6 might act as an exercise mimetic in skeletal muscles through the mechanisms involving the antioxidant system, inflammation, myogenesis, H_2_S signaling, mitochondrial function, the kynurenine pathway, and glycogenolysis (Table 1) [6]. Kumrungsee et al. found that adding B6 to a marginal B6-deficient diet increased the levels of antioxidant peptides, such as carnosine and anserine, in the heart and skeletal muscles of rodents [7,8] (Table 1). Suidasari et al. further reported that B6 supplementation increased the mRNA expression of nuclear factor erythroid 2-related factor 2 (Nrf2) and Nrf2-dependent antioxidant genes, such as heme oxygenase 1 (HO-1), superoxide dismutase 2 (SOD-2), glutathione peroxidase 1 (GPx-1), and glutathione S-transferase (GST), in skeletal muscles of rats [9]. Nrf2 is a component of the Kelch domain of Kelch-like ECH-associated protein 1 (Keap1)-Nrf2 pathway [10]. This pathway is the protective mechanism against oxidative and electrophilic stress by acting as a sensor that induces antioxidant gene expression. In addition to antioxidant responses, Nrf2 is involved in many other cellular processes, including metabolism, inflammation, and DNA instability [10,11,12]. Under normal conditions, Keap1 (a negative regulator of Nrf2 signaling) binds Nrf2, targeting it for degradation. Under stress conditions, Keap1 releases Nrf2, allowing it to initiate the gene expression of protective, antioxidant, and detoxification enzymes. Recently, several B6-treated animal models and cell cultures have demonstrated activation of Nrf2 signaling by showing increased expression of Nrf2-dependent genes and proteins and increased Nrf2 transactivation to the nucleus [13,14,15,16,17,18]. The detailed mechanism is, however, largely unclear. Of note, carnosine, anserine, and β-alanine are also activators of Nrf2 signaling in several animal models and cell cultures [19,20,21,22,23,24,25]. A recent study proposed the potential of targeted Nrf2 signaling regulation in the treatment of aging-related musculoskeletal, cardiac, and brain diseases [26,27,28].

**Figure 1:**
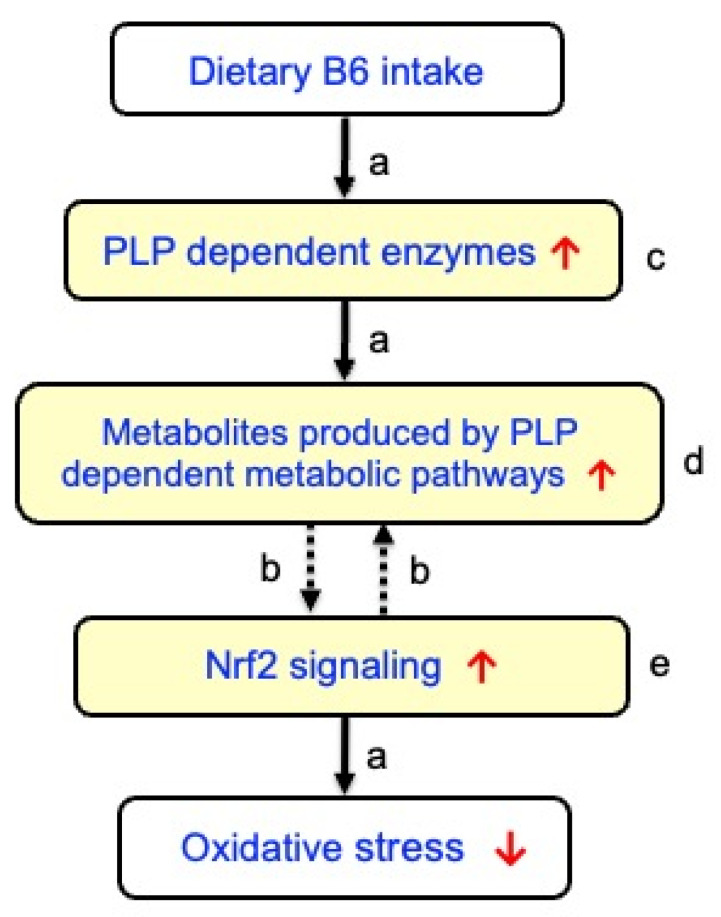
Potential role of B6 as an antioxidant mediated by orchestrating PLP-dependent multi-metabolic pathways and subsequent activation of Nrf2 signaling (hypothesis). (**a**) solid arrows indicating established mechanisms, (**b**) dotted arrows indicating suggestive (hypothetical) mechanisms, (**c**) antioxidant metabolites generated through PLP-dependent pathways (carnosine, anserine, taurine, H_2_S, 5-MTHF, KYNA, 3-HAA, and GABA), (**d**) PLP-dependent enzymes involved in the production of the antioxidant metabolites, and (**e**) Nrf2-dependent gene expression (HO-1, GST, GPx, SOD, GCL, NQO1, and xCT).

In view of these studies, we postulated that B6 supplementation might play beneficial roles mediated by increased PLP-dependent production of amino acid-derived metabolites, which in turn activate Nrf2 signaling (Table 1, Figure 1). To test this hypothesis, a survey of relevant studies in PubMed, Web of Science, and Google Scholar spanning publications from 1970 onward was conducted. Various combinations of terms were explored, such as vitamin B6, PLP, Nrf2, inflammation, oxidative stress, carnosine, taurine, skeletal muscle, H_2_S, energy metabolism, kynurenine pathway, and neurotransmitters. This review offers novel insights into the antioxidant role of B6 mediated by the production of several PLP-dependent metabolites.

## 2. Imidazole Peptides

Kato et al. revealed that compared with a low B6 diet (1 mg PN HCl/kg diet; marginal B6 deficiency level), a diet with the recommended B6 intake (7 mg PN HCl/kg diet, normal level) for 6 weeks markedly increased the levels of imidazole peptides, carnosine (β-alanyl-L-histidine) and anserine (β-alanyl-3-methylhistidine) in the heart and skeletal muscle of rodents [7,8] (Table 1). Notably, β-alanine, a precursor of carnosine and anserine, also significantly increased, whereas ornithine decreased sharply [7,8]. Ornithine can be metabolized to polyamines by ornithine decarboxylase (a PLP enzyme), which is then converted to β-alanine. Therefore, B6 supplementation could increase the metabolism from ornithine to carnosine, a naturally occurring histidine-containing peptide found in high concentrations in mammalian skeletal and cardiac muscles (Table 1). There is another piece of evidence suggesting that glutamate decarboxylase-like protein 1 (GADL1, a PLP enzyme) may also be responsible for β-alanine production by decarboxylating aspartic acid [29,34] (Table 1). The importance of GADL1 in carnosine synthesis was supported by human genetic studies showing a strong association of *GADL1* variants with blood levels of carnosine [29,34] However, further study is necessary to investigate whether B6 supplementation can increase GADL1 activity, thereby elevating β-alanine and carnosine production.

Carnosine has various beneficial properties, including its effect on muscle contraction, as well as antioxidant, anti-inflammatory, anti-glycation, anti-aging, and pH-buffering functions [35]. Carnosine, anserine, and β-alanine can modulate the endogenous antioxidant system by activating the signaling pathway controlled by the Nrf2 signaling [19,20,21,22,23,24,25], which is involved in the removal and detoxification of oxidative modification products of biomolecules and mitigating DNA damage. Interestingly, anserine binds to Keap1 with a binding force of −7.2 kcal/mol, increasing protein expressions of Nrf2, quinone oxidoreductase 1 (NQO1), and HO-1 in tert-butyl hydroperoxide (TBHP)-induced L-02 cells (a normal human liver cell line) exposed to anserine [24]. The detailed mechanism of activation of Nrf2 by carnosine remains to be clarified. A recent study indicated an inverse association between carnosine and sarcopenia progression in older adults [36]. Notably, carnosine modulates telomerase activity, slows down cell senescence [37], and affects the resistance of proteins to heat or chemical stress [38]. Therefore, the high levels of carnosine resulting from B6 supplementation may serve as a protective factor against sarcopenia and aging (Figure 1). However, further studies are necessary to explore whether the high carnosine levels resulting from B6 supplementation may be protective against frailty, sarcopenia, and aging through the Nrf2 signaling pathway.

## 3. Taurine

In mammals, the sulfur-containing amino acid taurine (2-aminoethanesulfonic acid) is the most abundant free amino acid. Komaru et al. reported that supplemental B6 to a marginal B6-deficient diet markedly increased the levels of taurine and hypotaurine in the skeletal muscle of mice (Table 1) [8]. In mammalian tissues, taurine is synthesized from cysteine via a three-step pathway involving cysteine oxidation to cysteine sulfinate, decarboxylation to hypotaurine by cysteine sulfinic acid decarboxylase (CSAD) (a PLP enzyme, Table 1), and final oxidation to taurine [39]. Taurine exerts multiple physiological functions, such as osmoregulation, pH regulation, antioxidative protection, and neuromodulation in mammals [40,41,42]. Taurine can also be obtained from foods, such as meat, fish, dairy products, and energy drinks, and is taken up by cells through taurine transporters. In humans, lower levels of taurine pathway metabolites were associated with various age-associated diseases, such as cardiovascular diseases, diabetes, and inflammation [43]. Recent studies have provided evidence suggesting the suppressed taurinylation of mitochondrial tRNAs during aging and mitochondrial dysfunction, a prominent feature of aging [44]. Taurine administration counteracted the aging-associated impingement of skeletal muscle regeneration by reducing inflammation and oxidative stress [45]. Agca et al. suggested that taurine activates the Nrf2 signaling pathway and attenuates the severity of oxidative stress by increasing Nrf2 and HO-1 expression levels in diabetic rats [46]. Treatment with hypotaurine promoted longevity and stress tolerance via the stress response factors such as Dauer formation-16 (DAF-16)/Forkhead box O (FOXO) and Skinhead-1 (SKN-1)/NRF2 in *Caenorhabditis elegans* (*C. elegans*) [47]. Xu et al. further reported that taurine treatment protected porcine mammary epithelial cells against H_2_O_2_-triggered oxidative stress by activating Nrf2 and scavenging ROS in a mitogen-activated protein kinase (MAPK)-dependent fashion [48]. Additionally, taurine supplementation promoted protein synthesis and proliferation of C2C12 myoblast cells through the mTOR signaling pathway, which is upregulated by Nrf2 [49]. Taken together, higher B6 intake may upregulate cysteine sulfinic acid decarboxylase, leading to increased taurine levels and improved aging-associated muscle dysfunction and oxidative stress (Figure 1).

## 4. H_2_S

H_2_S has emerged as a vital gasotransmitter, alongside nitric oxide and carbon monoxide, playing different roles in cellular signaling [50,51]. H_2_S is produced by the PLP enzyme, cystathionine-γ-lyase (CGL) (Table 1). Consequently, a lower B6 status is associated with reduced H_2_S levels [30,52]. H_2_S is involved in numerous physiological processes, such as vasodilation, neurotransmission, and cytoprotection [50,51]. Additionally, H_2_S modulates mitochondrial function, which promotes efficient energy production. H_2_S plays a dual role in physiological and pathological processes, particularly in the gastrointestinal tract. While physiological levels of H_2_S exert cytoprotective effects, excessive concentrations can lead to toxicity, oxidative stress, and inflammation [53]. Notably, H_2_S activates the Nrf2 signaling pathway via *S*-sulfhydration of Keap1, thereby promoting the expression of antioxidant enzymes such as GPx and SOD and protecting against cellular senescence [54]. Furthermore, H_2_S is crucial in regulating inflammation by inhibiting the production of proinflammatory cytokines, such as tumor necrosis factor-α (TNF-α) and interleukin-6 (IL-6), with levels increased during intense exercise, contributing to muscle soreness and injury [55]. Available evidence further suggests an essential role of H_2_S in maintaining myogenesis, presenting it as a potential candidate for the prevention of age-related sarcopenia and treatment of muscle injury [56]. Accordingly, B6 supplementation might be beneficial for health by increasing H_2_S levels (Figure 1). Furthermore, Wang et al. reported notable findings that exogenous H_2_S promoted E3 ligase synoviolin (Syvn1)-mediated Keap1 ubiquitination, leading to the suppression of the Nrf2 signaling pathway in *db*/*db* mice with diabetic cardiomyopathy [57].

## 5. Supersulfides

Recently, Zhang et al. proposed “supersulfides” to describe species with catenated sulfur moieties, such as hydropersulfides (RSSH), hydropolysulfides (RSSnH, n > 1), polysulfides (RSSnR, n > 1), and inorganic persulfides [58]. A recent breakthrough involves the identification of PLP-dependent supersulfide biosynthetic enzymes, such as cysteinyl-tRNA synthetase (CARS), which constitute a crucial pathway for energy metabolism in cells [31] (Table 1). Additionally, the sensitivity of CARS to B6 deficiency has been reported [31]. Like other aminoacyl-tRNA synthetases, CARS is primarily involved in cysteinyl-tRNA production utilizing cysteine. Supersulfides have several physiological functions, such as acting as potent antioxidants, regulating redox signaling, and influencing energy production, inflammation, and cellular processes [59]. However, whether supersulfides activate Nrf2 signaling remains unclear and requires further study.

## 6. 5-Methyltetrahydrofolate

The production of 5-methyltetrahydrofolate (5-MTHF), the primary bioactive and circulating form of folate, occurs through a multi-step pathway. Tetrahydrofolate (THF) is first converted into 5,10-methylenetetrahydrofolate (5,10-MTHF) by a PLP-dependent enzyme, serine hydroxymethyltransferase (SHMT) [60] (Table 1). Subsequently, 5,10-MTHF is converted into 5-MTHT by methylenetetrahydrofolate reductase (MTHFR). There are some reports of the liver SHMT activity being reduced by a marginal B6 deficiency [32]. This reduction was associated with reduced 5-MTHF levels. A study indicated that injection of 5-MTHF activated the Nrf2 signaling and increased the expression of GCL, SOD-1, and HO-1 in the kidneys of rats that developed acute kidney injury [61]. A recent study further reported that the treatment with 5-MTHF exerted anti-inflammatory effects in RAW264.7 cells and zebrafish [62]. Thus, supplemental B6 might exert a beneficial effect on health by activating Nrf2 signaling caused by increasing 5-MTHF (Figure 1).

## 7. Kynurenine Pathway Metabolites

The tryptophan-kynurenine pathway is the primary metabolic route for breaking down the amino acid tryptophan into various neuroactive and immunomodulatory compounds, such as kynurenine, kynurenic acid (KYNA), and 3-hydroxyanthranillic acid (3-HAA) [63,64] (Table 1). This pathway is crucial for the functioning of the nervous, immune, and endocrine systems and is implicated in various diseases, including inflammatory and neurodegenerative diseases, as well as frailty, sarcopenia, and osteoporosis [63,64]. In the kynurenine pathway, KYNA and 3-hydroxyanthranic acid are produced by PLP enzymes, kynurenine aminotransferase (KAT) and kynureninase (KYNU), respectively (Table 1). In a human clinical study, consumption of PN (40 mg/day) for one month significantly increased plasma PLP, KYNA, and 3HAA levels in cardiovascular patients (Table 1) [33]. KYNA and 3-HAA can function as antioxidants by scavenging free radicals, such as hydroxyl radicals, superoxide, and peroxynitrite [65,66]; increase the activity of cellular antioxidant enzymes, such as SOD, CAT, and GPx, through the Nrf2 signaling pathway; and suppress pro-inflammatory pathways, such as those involving NF-κB and pro-inflammatory cytokines [67]. A recent study with *C. elegans* suggested that 3-HAA may have antiaging effects and extend healthy lifespan through upregulating Nrf2 signaling [68]. Thus, supplemental B6 might exert a beneficial impact by activation of the Nrf2 signaling (Figure 1).

## 8. GABA

γ-Aminobutyrate (GABA) is a naturally occurring neurotransmitter synthesized from glutamic acid through the enzymatic action of the PLP enzyme glutamic acid decarboxylase [69]. The GABA synthesis (GAD activity) by skin fibroblasts from an infant with pyridoxine-dependent seizures was reduced compared with controls [70]. PLP-independent GAD activity was similar in control and patient fibroblasts, whereas the patient’s PLP-dependent GAD activity was reduced compared with controls. Supplemental B6 added to a marginal B6-deficient diet increased cardiac GABA levels in rats [7] (Table 1). Animal studies have linked age-related reductions in the levels of GABA, the primary inhibitory neurotransmitter, to age-related cognitive, motor, and sensory decline [71]. Jin et al. further suggested that GABA might have potential uses in preventing age-related sarcopenic obesity and related metabolic diseases [72]. Moreover, GABA has various biological activities such as its antioxidant, anti-inflammatory, stress-alleviating, and sleep-promoting properties [73,74,75]. Horii et al. reported that high-dose GABA supplementation in diabetic mice during muscle regeneration elevated circulating GABA levels but delayed muscle repair, possibly due to its antioxidant and anti-inflammatory effects during the early phase of injury, when inflammation and phagocyte activity are essential for effective regeneration [76]. In contrast, low-dose supplementation of GABA appeared to promote muscle regeneration. These findings suggest that GABA might exert dose-dependent dual effects in physiological processes in which early inflammatory responses play a critical role, such as muscle repair. Notably, a recent study with *C. elegans* indicated that the GABA treatment activated the Nrf2 signaling pathway [77].

## 9. Glycogenolysis

**Figure 2:**
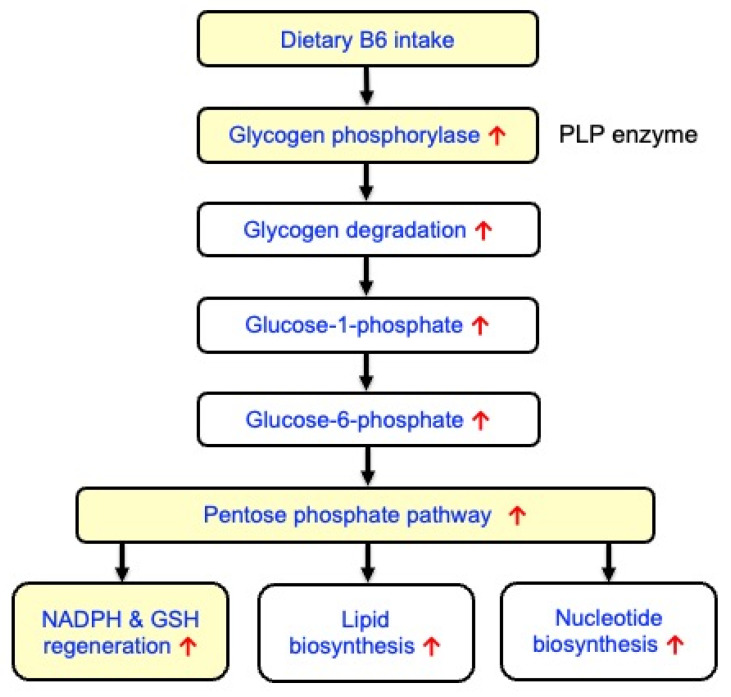
Supplemental B6 upregulates glycogen phosphorylase and the pentose phosphate pathway (PPP), and subsequent regeneration of NADPH and GSH in skeletal muscle.

Skeletal muscle tissue is the primary storage site for B6, holding 80–90% of the body’s total pool, predominantly as PLP bound to glycogen phosphorylase (GP). PLP serves as a cofactor for GP, an enzyme crucial for muscle glycogen breakdown (glycogenolysis) [78,79] (Figure 2). Okada et al. indicated that B6 deficiency reduced the GP activity in rat skeletal muscles [80]. B6 supplementation increases the GP activity, which can enhance muscle glycogen degradation and utilization, potentially improving physical performance and exercise capacity. In skeletal muscle, stimulation of glycogenolysis may redirect glucose flux from glycolysis to the pentose phosphate pathway (PPP), playing a crucial role in maintaining cellular redox balance and providing essential precursors for de novo synthesis of lipids and nucleotides (Figure 2). For reducing oxidative stress, the PPP produces nicotinamide adenine dinucleotide phosphate (NADPH) and thus regenerates GSH through NADPH-linked redox systems [81] (Figure 2). Furthermore, Nrf2 signaling promoted gene expression of NADPH-generating enzymes, such as glucose 6-phosphate dehydrogenase and 6-phosphogluconate dehydrogenase [82,83]. Thus, B6 intake might be protective against oxidative stress in skeletal muscles by stimulating glycogen breakdown and regeneration of NADPH and GSH, as well as Nrf2 signaling activation (Figure 2).

## 10. Overall Discussion

This study aimed to test the hypothesis that B6 supplementation may exert potential antioxidant effects via PLP-dependent multi-metabolic modulation (Figure 1). To this end, relevant literature was reviewed. Overall, the analyzed studies appear to support our hypothesis. Beyond its direct role as an antioxidant, B6 could contribute to key antioxidant defenses that drive changes across different antioxidant-producing pathways. The beneficial effect of B6 might be partly mediated by the mechanisms through the activation of Nrf2 signaling. However, it is still possible that B6 might activate Nrf2 signaling via mechanisms independent of amino acid metabolism. Furthermore, the proposed pathway (Figure 1) is supported by separate lines of evidence (Table 1). Therefore, this study does not necessarily imply that the mechanisms of action of various antioxidant metabolites converge into a unified pathway involving Nrf2.

Recently, Ryan et al. reported significantly higher levels of carnosine, β-alanine, taurine, hypotaurine, and GSH in wild-type macrophages than in Nrf2-knockout macrophages [84]. These findings are similar to those observed in B6-treated animals. Hence, B6-induced increases in the antioxidant metabolites might activate Nrf2 signaling, which could further increase the antioxidant metabolites, forming a positive feedback loop.

This study further suggests that dietary protein restriction might reduce endogenous production of amino acid–derived antioxidants and subsequently disrupt Nrf2 signaling. Consistent with this hypothesis, Hruby et al. reported the inverse associations of low protein intake with longitudinal changes in oxidative stress biomarkers over the long term in a community-dwelling population [85]. Recently, He et al. have reported that dietary protein and some amino acids and peptides, such as branched-chain amino acids, arginine, glutamine, glycine, serine, taurine, β-alanine, and carnosine, could help mitigate sarcopenia in humans [86]. Thus, adequate intake of proteins and amino acids may help support metabolic pathways linked to B6-induced activation of Nrf2 signaling.

Intriguingly, Varghese et al. reported that cysteine restriction, by depleting GSH, intensively affected weight loss, metabolism, and stress signaling compared with other amino acid restrictions [87]. As shown in Table 1, cysteine and cystathionine (converted to cysteine) are metabolized to specific sulfur-containing antioxidants, such as taurine, hypotaurine, H_2_S, and supersulfides. Therefore, the conversion of cysteine into several key antioxidants might, at least in part, explain the pronounced effect of cysteine restriction. Additionally, Nrf2 signaling upregulates the gene expression of xCT (cystine transporter, SLC7A11) [88]. Hence, Nrf2 may contribute to the positive regulation of cysteine uptake and metabolism.

This study suggests a potential association of B6 deficiency with chronic diseases via Nrf2 signaling. However, these associations might reflect overall nutritional status related to the changes in B6 and Nrf2 signaling as confounding factors. Furthermore, modulations of oxidative stress and inflammation might affect the B6 status and Nrf2 signaling as reverse causation.

## 11. Conclusions and Further Studies

To the best of our knowledge, this study provides a novel insight into the antioxidant mechanism of B6, which may, at least partially, involve the PLP-dependent multi-metabolic production of several antioxidants and subsequent activation of Nrf2 signaling. As a practical implication of our hypothesis, this hypothesis implies that exploring nutrients and endogenous metabolites that activate Nrf2 is a promising, evidence-based approach for preventing chronic oxidative stress and age-related diseases.

However, the central limitation of the paper lies heavily in this analysis of indirect and heterogeneous evidence while presenting a highly integrative mechanistic model that remains largely hypothetical. Furthermore, there is no direct evidence demonstrating this full cascade of the model. Additionally, most of our discussions are based on animal experiments, and many of the findings are not verified in humans. Thus, human clinical investigations are needed to clarify the relationship between dietary B6 intake and the antioxidant metabolites analyzed and to assess the proposed pathway (Figure 1).

Recent accumulating evidence has highlighted a crucial role of B6 in regulating signaling networks in oxidative stress and inflammation through the mechanisms involving NF-κB and NLRP3 inflammasomes [1,5]. Similarly, B6 may play an important role in the regulation of oxidative stress response and inflammation via Nrf2. Therefore, further study is of great interest to elucidate the detailed roles of B6 through Nrf2 signaling. In particular, the interrelationship between the antioxidant metabolites discussed herein and Nrf2 signaling remains unclear, and mechanistic studies should be focused on this point. Finally, the proposed framework in this article is conceptual and intended to stimulate future research, rather than to describe a validated mechanistic pathway.

## Figures and Tables

**Table 1:** PLP-dependent enzymes are needed to produce antioxidant metabolites.

Substrates for a PLP Dependent Metabolism	PLP Enzymes Needed to Produce Antioxidant Metabolites	Metabolites Produced by PLP-Dependent Metabolic Pathways	Physiological and Translational Relevance
ornithine	ornithine decarboxylase (ODC)	carnosine and anserine	Supplemental B6 at a physiological level for a marginal B6-deficient diet (1 mg PN HCl/kg) for 6 weeks increased carnosine and anserine levels and decreased ornithine levels in skeletal muscles of rats (animal study) [7] *.
aspartic acid	glutamate decarboxylase-like protein 1 (GADL1)	carnosine and anserine	Gadl1 knockout mice were deficient in carnosine in skeletal muscle (animal study) [29].
cysteine	cysteine sulfinic acid decarboxylase (CSAD)	taurine	Supplemental B6 at a physiological level for a marginal B6-deficient diet for 6 weeks increased taurine levels in the skeletal muscle of mice (animal study) [8].
cystathionine	cystathionine-γ-lyase (CGL)	hydrogen sulfide (H_2_S)	Cell culture in B6-deficient medium for 6 weeks reduced levels of H_2_S in HepG2 cells (cell culture study) [30].
cysteine	cysteinyl-tRNA synthetase (CARS)	supersulfides	The addition of PLP at physiological levels increased the production of supersulfides (in vitro study) [31].
5,10-methylenetetrahydrofolate (5,10-MTHF)	serine hydroxymethyl transferase (SHMT)	5-methyltetrahydrofolate (5-MTHF)	Intake of a marginal B6-deficient diet (0.5 mg PN/kg) for 35 days decreased liver SHMT activity in rats (animal study) [32].
kynurenine (KYN)	kynurenine aminotransferase (KAT)	kynurenic acid (KYNA)	Consumption of PN (40 mg/day) for one month significantly increased plasma PLP and KYNA levels in cardiovascular patients (human study) [33].
3-hydroxykynurenine (3-HK)	kynureninase (KYNU)	3-hydroxyanthranilic acid (3-HAA)	Consumption of a supplementary dose of PN (40 mg/day) for one month significantly increased plasma 3-HAA levels in cardiovascular patients (human study) [33].
glutamic acid	glutamic acid decarboxylase (GAD)	γ-aminobutyric acid (GABA)	Supplemental B6 at a physiological level for a marginal B6-deficient diet for 6 weeks increased cardiac GABA levels in rats (animal study) [7].

* Numbers in brackets indicate references.

## Data Availability

No new data were created in this study.

## References

[1] Stach K., Stach W., Augoff K. (2021). Vitamin B6 in Health and Disease. Nutrients.

[2] Troesch B., Hoeft B., McBurney M., Eggersdorfer M., Weber P. (2012). Dietary surveys indicate vitamin intakes below recommendations are common in representative Western countries. Br. J. Nutr..

[3] Wang P., Huang J., Xue F., Abuduaini M., Tao Y., Liu H. (2024). Associations of serum vitamin B6 status with the risks of cardiovascular, cancer, and all-cause mortality in the elderly. Front. Immunol..

[4] Tong T., Quan H., Kim C.K., Zeng W. (2024). Editorial: Role of nutrition in skeletal muscle atrophy and sarcopenia. Front. Nutr..

[5] Kato N., Kimoto A., Zhang P., Bumrungkit C., Karunaratne S., Yanaka N., Kumrungsee T. (2024). Relationship of Low Vitamin B6 Status with Sarcopenia, Frailty, and Mortality: A Narrative Review. Nutrients.

[6] Kato N., Yang Y., Bumrungkit C., Kumrungsee T. (2024). Does Vitamin B6 Act as an Exercise Mimetic in Skeletal Muscle?. Int. J. Mol. Sci..

[7] Kumrungsee T., Nirmagustina D.E., Arima T., Onishi K., Sato K., Kato N., Yanaka N. (2019). Novel metabolic disturbances in marginal vitamin B6-deficient rat heart. J. Nutr. Biochem..

[8] Komaru T., Yanaka N., Kumrungsee T. (2021). Satellite Cells Exhibit Decreased Numbers and Impaired Functions on Single Myofibers Isolated from Vitamin B6-Deficient Mice. Nutrients.

[9] Suidasari S., Uragami S., Yanaka N., Kato N. (2017). Dietary vitamin B6 modulates the gene expression of myokines, Nrf2-related factors, myogenin and HSP60 in the skeletal muscle of rats. Exp. Ther. Med..

[10] Saha S., Buttari B., Saso L. (2020). An Overview of Nrf2 Signaling Pathway and Its Role in Inflammation. Molecules.

[11] Li J., Xu C., Liu Q. (2023). Roles of NRF2 in DNA damage repair. Cell. Oncol..

[12] Ham S., Choi B.H., Kwak M.K. (2024). NRF2 signaling and amino acid metabolism in cancer. Free Radic. Res..

[13] Li C., Wang R., Hu C., Wang H., Ma Q., Chen S., He Y. (2018). Pyridoxine exerts antioxidant effects in cell model of Alzheimer’s disease via the Nrf-2/HO-1 pathway. Cell. Mol. Biol..

[14] Wei Y., Lu M., Mei M., Wang H., Han Z., Chen M., Yao H., Song N., Ding X., Ding J. (2020). Pyridoxine induces glutathione synthesis via PKM2-mediated Nrf2 transactivation and confers neuroprotection. Nat. Commun..

[15] Shan M., Yu X., Li Y., Fu C., Zhang C. (2021). Vitamin B6 Alleviates Lipopolysaccharide-induced Myocardial Injury by Ferroptosis and Apoptosis Regulation. Front. Pharmacol..

[16] Ruan R., Shao W., Su Y., Liu J., Luo J., Luo Y., Wang L., Fan X. (2024). Early pyridoxine administration rescues autism-like behavior in the BTBR T+tf/J autistic model. Res. Autism Spectr. Disord..

[17] Nasir A., Rahman M.U., Khan M., Zahid M., Shahab M., Jiao H., Zeb A., Shah A.S., Khan H. (2025). Vitamin B6 via p-JNK/Nrf-2/NF-κB Signaling Ameliorates Cadmium Chloride-Induced Oxidative Stress Mediated Memory Deficits in Mice Hippocampus. Curr. Neuropharmacol..

[18] Jaruan O., Promsan S., Thongnak L., Pengrattanachot N., Phengpol N., Sutthasupha P., Lungkaphin A. (2025). Pyridoxine exerts antioxidant effects on kidney injury manifestations in high-fat diet-induced obese rats. Chem. Biol. Interact..

[19] Zhang S., Li Y., Liu X., Guo S., Jiang L., Huang Y., Wu Y. (2023). Carnosine alleviates kidney tubular epithelial injury by targeting NRF2 mediated ferroptosis in diabetic nephropathy. Amino Acids.

[20] Palin M.-F., Lapointe J., Gariépy C., Beaudry D., Kalbe C. (2020). Characterisation of intracellular molecular mechanisms modulated by carnosine in porcine myoblasts under basal and oxidative stress conditions. PLoS ONE.

[21] Caruso G., Privitera A., Antunes B.M., Lazzarino G., Lunte S.M., Aldini G., Caraci F. (2022). The Therapeutic Potential of Carnosine as an Antidote against Drug-Induced Cardiotoxicity and Neurotoxicity: Focus on Nrf2 Pathway. Molecules.

[22] Meftahi G.H., Jahromi G.P. (2023). Biochemical Mechanisms of Beneficial Effects of Beta-Alanine Supplements on Cognition. Biochemistry.

[23] Busa P., Lee S.-O., Huang N., Kuthati Y., Wong C.-S. (2022). Carnosine Alleviates Knee Osteoarthritis and Promotes Synoviocyte Protection via Activating the Nrf2/HO-1 Signaling Pathway: An In-Vivo and In-Vitro Study. Antioxidants.

[24] Chen M., Luo J., Ji H., Song W., Zhang D., Su W., Liu S. (2023). The Preventive Mechanism of Anserine on Tert-Butyl Hydroperoxide-Induced Liver Injury in L-02 Cells via Regulating the Keap1-Nrf2 and JNK-Caspase-3 Signaling Pathways. Mar. Drugs.

[25] He H., Lv C., Xie Y., Li W., Ling Z., Cheng B., Tao X. (2025). Carnosine alleviates oxidative stress to prevent cellular senescence by regulating Nrf2/HO-1 pathway: A promising anti-aging strategy for oral mucosa. Front. Pharmacol..

[26] Zhanga X., Li H., Chen L., Wu Y., Yusheng Li Y. (2024). NRF2 in age-related musculoskeletal diseases: Role and treatment prospects. Genes Dis..

[27] Khan S.U., Shahid Khan S.U., Suleman M., Khan M.U., Khan M.S., Arbi F.M., Hussain T., Alsuhaibani A.M. (2024). Natural Allies for Heart Health: Nrf2 Activation and Cardiovascular Disease Management. Curr. Probl. Cardiol..

[28] Amoroso R., Maccallini C., Bellezza I. (2023). Activators of Nrf2 to Counteract Neurodegenerative Diseases. Antioxidants.

[29] Mahootchi E., Homaei S.C., Kleppe R., Winge I., Hegvik T.-A., Megias-Perez R., Totland C., Mogavero F., Baumann A., Glennon J.C. (2020). GADL1 is a multifunctional decarboxylase with tissue-specific roles in β-alanine and carnosine production. Sci. Adv..

[30] Deratt B.N., Ralat M.A., Kabil O., Chi Y.-Y., Banerjee R., Gregory J.F. (2014). Vitamin B-6 Restriction Reduces the Production of Hydrogen Sulfide and its Biomarkers by the Transsulfuration Pathway in Cultured Human Hepatoma Cells. J. Nutr..

[31] Akaike T., Ida T., Wei F.-Y., Nishida M., Kumagai Y., Alam M.M., Ihara H., Sawa T., Matsunaga T., Kasamatsu S. (2017). Cysteinyl-tRNA synthetase governs cysteine polysulfidation and mitochondrial bioenergetics. Nat. Commun..

[32] Scheer J.B., Mackey A.D., Gregory J.F. (2005). Activities of hepatic cytosolic and mitochondrial forms of serine hydroxymethyl- transferase and hepatic glycine concentration are affected by vitamin B-6 intake in rats. J. Nutr..

[33] Midttun Ø., Ulvik A., Pedersen E.R., Ebbing M., Bleie Ø., Schartum-Hansen H., Nilsen R.M., Nygard O., Ueland P.M. (2011). Low Plasma Vitamin B-6 Status Affects Metabolism through the Kynurenine Pathway in Cardiovascular Patients with Systemic Inflammation. J. Nutr..

[34] Shin S.-Y., Fauman E.B., Petersen A.-K., Krumsiek J., Santos R., Huang J., Arnold M., Erte I., Forgetta V., Yang T.-P. (2014). An atlas of genetic influences on human blood metabolites. Nat. Genet..

[35] Solana-Manrique C., Sanz F.J., Martínez-Carrión G., Paricio N. (2022). Antioxidant and Neuroprotective Effects of Carnosine: Therapeutic Implications in Neurodegenerative Diseases. Antioxidants.

[36] Hsu W.-H., Wang A.-Y., Chao Y.-M., Chang K.-V., Han D.-S., Lin Y.-L. (2024). Novel metabolic and lipidomic biomarkers of sarcopenia. J. Cachexia Sarcopenia Muscle.

[37] Holliday R., McFarland G.A. (2000). A role for carnosine in cellular maintenance. Biochem. (Mosc.).

[38] Villari V., Attanasio F., Micali N. (2014). Control of the Structural Stability of α-Crystallin under Thermal and Chemical Stress: The Role of Carnosine. J. Phys. Chem. B..

[39] Mahootchia E., Raasakkaa A., Luan W., Muruganandam G., Loris R., Haavik J., Kursula P. (2021). Structure and substrate specificity determinants of the taurine biosynthetic enzyme cysteine sulphinic acid decarboxylase. J. Struct. Biol..

[40] Santulli G., Kansakar U., Varzideh F., Mone P., Jankauskas S.S., Lombardi A. (2023). Functional Role of Taurine in Aging and Cardiovascular Health: An Updated Overview. Nutrients.

[41] Schaffer S., Kim H.W. (2018). Effects and Mechanisms of Taurine as a Therapeutic Agent. Biomol. Ther..

[42] Seidel U., Huebbe P., Rimbach G. (2019). Taurine: A regulator of cellular redox homeostasis and skeletal muscle function. Mol. Nutr. Food Res..

[43] Singh P., Gollapalli K., Mangiola S., Schranner D., Yusuf M.A., Chamoli M., Shi S.L., Riermeier A., Vayndorf E.M., Riermeier A. (2023). Taurine deficiency as a driver of aging. Science.

[44] Fakruddin M., Wei F.-Y., Suzuki T., Asano K., Kaieda T., Omori A., Izumi R., Fujimura A., Kaitsuka T., Miyata K. (2018). Defective Mitochondrial tRNA Taurine Modification Activates Global Proteostress and Leads to Mitochondrial Disease. Cell Rep..

[45] Barbiera A., Sorrentino S., Fard D., Lepore E., Sica G., Dobrowolny G., Tamagnone L., Scicchitano B.M. (2022). Taurine Administration Counteracts Aging-Associated Impingement of Skeletal Muscle Regeneration by Reducing Inflammation and Oxidative Stress. Antioxidants.

[46] Agca C.A., Tuzcu M., Hayirli A., Sahin K. (2014). Taurine Ameliorates Neuropathy via Regulating NF-κB and Nrf2/HO-1 Signaling Cascades in Diabetic Rats. Food Chem. Toxicol..

[47] Wan Q.-L., Fu X., Meng X., Luo Z., Dai W., Yang J., Wang C., Wang H., Zhou Q. (2020). Hypotaurine promotes longevity and stress tolerance *via* the stress response factors DAF-16/FOXO and SKN-1/NRF2 in *Caenorhabditis elegans*. Food Funct..

[48] Xu M., Che L., Gao K., Wang L., Yang X., Wen X., Li M., Jiang Z. (2023). Taurine alleviates oxidative stress in porcine mammary epithelial cells by stimulating the Nrf2- MAPK signaling pathway. Food Sci. Nutr..

[49] Hao Q., Wang L., Zhang M., Wang Z., Li M., Gao X. (2022). Taurine stimulates protein synthesis and proliferation of C2C12 myoblast cells through the PI3K-ARID4B-mTOR pathway. Br. J. Nutr..

[50] Cirino G., Szabo C., Papapetropoulos A. (2023). Physiological roles of hydrogen sulfide in mammalian cells, tissues, and organs. Physiol. Rev..

[51] Shahid A., Bhatia M. (2024). Hydrogen Sulfide: A Versatile Molecule and Therapeutic Target in Health and Diseases. Biomolecules.

[52] Mys L., Goshovska Y., Strutynska N., Fedichkina R., Korkach Y., Strutynskyi R., Sagach V. (2022). Pyridoxal-5-phosphate induced cardioprotection in aging associated with up-expression of cystathionine-γ-lyase, 3-mercaptopyruvate sulfurtransferase, and ATP-sensitive potassium channels. Eur. J. Clin. Investig..

[53] Mallardi D., Chimienti G., Maqoud F., Orlando A., Drago S., Malerba E., De Virgilio C., Akbarali H.I., Russo F. (2025). The Dual Role of Exogenous Hydrogen Sulfide (H_2_S) in Intestinal Barrier Mitochondrial Function: Insights into Cytoprotection and Cytotoxicity Under Non-Stressed Conditions. Antioxidants.

[54] Yang G., Zhao K., Ju Y., Mani S., Cao Q., Puukila S., Khaper N., Wu L., Wang R. (2013). Hydrogen Sulfide Protects Against Cellular Senescence *via S*-Sulfhydration of Keap1 and Activation of Nrf2. Antioxid. Redox Signal..

[55] Zhang Y., Masters L., Wang Y., Wu L., Pei Y., Guo B., Parissenti A., Lees S.J., Wang R., Yang G. (2021). Cystathionine gamma-lyase/H_2_S signaling facilitates myogenesis under aging and injury condition. FASEB J..

[56] Yang J.-H., Gao J., E Y.-Q., Jiao L.-J., Wu R., Yan Q.-Y., Wei Z.-Y., Yan G.-L., Liang J.-L., Li H.-Z. (2024). Hydrogen sulfide inhibits skeletal muscle ageing by up-regulating autophagy through promoting deubiquitination of adenosine 5′-monophosphate (AMP)-activated protein kinase α1 via ubiquitin specific peptidase 5. J. Cachexia Sarcopenia Muscle.

[57] Wang M., Tang J., Zhang S., Pang K., Zhao Y., Liu N., Huang J., Kang J., Dong S., Li H. (2023). Exogenous H_2_S initiating Nrf2/GPx4/GSH pathway through promoting Syvn1-Keap1 interaction in diabetic hearts. Cell Death Discov..

[58] Zhang T., Toyomoto T., Sawa T., Akaike T., Matsunaga T. (2025). Supersulfides: A Promising Therapeutic Approach for Autoinflammatory Diseases. Microbiol. Immunol..

[59] Nishimura A., Yoon S., Matsunagaa T., Ida T., Junga M., Ogata S., Morita M., Yoshitake J., Unno Y., Barayeua U. (2024). Longevity control by supersulfide-mediated mitochondrial respiration and regulation of protein quality. Redox Biol..

[60] Bajic Z., Sobot T., Skrbic R., Stojiljkovic M.P., Ponorac N., Matavulj A., Djuric D.M. (2022). Homocysteine, Vitamins B6 and Folic Acid in Experimental Models of Myocardial Infarction and Heart Failure—How Strong Is That Link?. Biomolecules.

[61] Wijerathne C.U.B., Au-Yeung K.K.W., Siow Y.L., O K. (2022). 5-Methyltetrahydrofolate Attenuates Oxidative Stress and Improves Kidney Function in Acute Kidney Injury through Activation of Nrf2 and Antioxidant Defense. Antioxidants.

[62] Bin X.-N., Gao Y.-B., Pan M., Lian Z.-L., Cheng Y.-Z., Wu J.-Q., He M.-F. (2023). Anti-inflammatory effects of 6S-5-methyltetrahydrofolate-calcium on RAW264.7 cells and zebrafish. Life Sci..

[63] Ballesteros J., Rivas D., Duque G. (2023). The Role of the Kynurenine Pathway in the Pathophysiology of Frailty, Sarcopenia, and Osteoporosis. Nutrients.

[64] Yang Y., Liu X., Liu X., Xie C., Shi J. (2024). The role of the kynurenine pathway in cardiovascular disease. Front. Cardiovasc. Med..

[65] Alves L.F., Moore J.B., Kell D.B. (2024). The Biology and Biochemistry of Kynurenic Acid, a Potential Nutraceutical with Multiple Biological Effects. Int. J. Mol. Sci..

[66] Leipnitza G., Schumachera C., Dalcina K.B., Scussiatoa K., Solano A., Funchal C., Dutra-Filhoa C.S., Wyse A.T.S., Wannmacher C.M.D., Latini A. (2007). In vitro evidence for an antioxidant role of 3-hydroxykynurenine and 3-hydroxyanthranilic acid in the brain. Neurochem. Int..

[67] Krause D., Suh H.-S., Tarassishin L., Cui Q.L., Durafourt B.A., Choi N., Bauman A., Cosenza-Nashat M., Antel J.P., Zhao M.-L. (2011). The Tryptophan Metabolite 3-Hydroxyanthranilic Acid Plays Anti-Inflammatory and Neuroprotective Roles During Inflammation Role of Hemeoxygenase-1. Am. J. Pathol..

[68] Dang H., Castro-Portuguez R., Espejo L., Backer G., Freitas S., Spence E., Meyers J., Shuck K., Gardea E.A., Chang L.M. (2023). On the benefits of the tryptophan metabolite 3-hydroxyanthranilic acid in *Caenorhabditis elegans* and mouse aging. Nat. Commun..

[69] Heli Z., Hongyu C., Dapeng B., Yee Shin T., Yejun Z., Xi Z., Yingying W. (2022). Recent advances of γ-aminobutyric acid: Physiological and immunity function, enrichment, and metabolic pathway. Front. Nutr..

[70] Gospe S.M., Olin K.L., Keen C.L. (1994). Reduced GABA synthesis in pyridoxine-dependent seizures. Lancet.

[71] Cuypers K., Maes C., Swinnen S.P. (2018). Aging and GABA. Aging.

[72] Jin H., Oh H.-J., Lee B.-Y. (2023). GABA Prevents Age-Related Sarcopenic Obesity in Mice with High-Fat-Diet-Induced Obesity. Cells.

[73] Hepsomali P., Groeger J.A., Nishihira J., Scholey A. (2020). Effects of Oral Gamma-Aminobutyric Acid (GABA) Administration on Stress and Sleep in Humans: A Systematic Review. Front. Neurosci..

[74] Fu J., Han Z., Wu Z., Xia Y., Yang G., Yulong Yin Y., Wenkai Ren W. (2022). GABA regulates IL-1b production in macrophages. Cell Rep..

[75] Zhu Z., Shic Z., Xie C., Gong W., Hu Z., Peng Y. (2019). A novel mechanism of Gamma-aminobutyric acid (GABA) protecting human umbilical vein endothelial cells (HUVECs) against H_2_O_2_-induced oxidative injury. Comp. Biochem. Physiol..

[76] Horii M., Bumrungkit C., Yanaka N., Hawke T.J., Rebalka I.A., Kumrungsee T. (2025). Effects of oral γ-aminobutyric acid intake on muscle regeneration in diabetic mice. Am. J. Physiol. Cell Physiol..

[77] Teng J., Ting Yu T., Yan F. (2024). GABA attenuates neurotoxicity of zinc oxide nanoparticles due to oxidative stress via DAF-16/FoxO and SKN-1/Nrf2 pathways. Sci. Total Environ..

[78] Migocka-Patrzałek M., Elias M. (2021). Muscle Glycogen Phosphorylase and Its Functional Partners in Health and Disease. Cells.

[79] Katz A. (2022). A century of exercise physiology: Key concepts in regulation of glycogen metabolism in skeletal muscle. Eur. J. Appl. Physiol..

[80] Okada M., Ishikawa K., Watanabe K. (1991). Effect of Vitamin B6 Deficiency on Glycogen Metabolism in the Skeletal Muscle, Heart, and Liver of Rats. J. Nutr. Sci. Vitaminol..

[81] Bradshaw P.C. (2019). Cytoplasmic and Mitochondrial NADPH-Coupled Redox Systems in the Regulation of Aging. Nutrients.

[82] Thimmulappa R.K., Biswal S., Mai K.H., Srisuma S., Kensler T.W., Yamamoto M., Biswal S. (2002). Identification of Nrf2-regulated genes induced by the chemopreventive agent sulforaphane by oligonucleotide microarray. Cancer Res..

[83] Song M.-Y., Lee D.-Y., Chun K.-S., Kim E.-H. (2021). The Role of NRF2/KEAP1 Signaling Pathway in Cancer Metabolism. Int. J. Mol. Sci..

[84] Ryan D.G., Knatko E.V., Casey A.M., Jens L., Hukelmann J., Naidu S.D., Brenes A.J., Ekkunagul T., Baker C., Higgins M. (2022). Nrf2 activation reprograms macrophage intermediary metabolism and suppresses the type I interferon response. iScience.

[85] Hruby A., Jacques P.F. (2019). Dietary Protein and Changes in Biomarkers of Inflammation and Oxidative Stress in the Framingham Heart Study Offspring Cohort. Curr. Dev. Nutr..

[86] He W., Connolly E.D., Cross H.R., Wu G. (2025). Dietary protein and amino acid intakes for mitigating sarcopenia in humans. Crit. Rev. Food Sci. Nutr..

[87] Varghese A., Gusarov I., Gamallo-Lana B., Dolgonos D., Mankan Y., Shamovsky I., Phan M., Jones R., Gomez-Jenkins M., White E. (2025). Unravelling cysteine-deficiency-associated rapid weight loss. Nature.

[88] Jyotsana N., Ta K.T., Dergiorno K.E. (2022). The Role of Cystine/Glutamate Antiporter SLC7A11/xCT in the Pathophysiology of Cancer. Front. Oncol..

